# Design and evaluation of child abuse web-based application for parent education & strengthen

**DOI:** 10.1186/s12889-024-18248-9

**Published:** 2024-03-05

**Authors:** Sharif Para, Hassan Shahrokhi, Elham Maserat, Zeinab Mohammadzadeh

**Affiliations:** 1 Department of Health Information Technology, Student Research Committee, School of Management and Medical Informatics, Tabriz University of Medical Sciences, Tabriz, Iran; 2 Research Center of Psychiatry and Behavioral Sciences, Tabriz University of Medical Sciences, Tabriz, Iran; 3 Department of Medical Informatics, Faculty of Medical Sciences, Tarbiat Modares University, Tehran, Iran; 4 Department of Health Information Technology, School of Management and Medical Informatics, Tabriz University of Medical Sciences, Daneshgah St, 5165665811 Tabriz, Iran

**Keywords:** Child abuse prevention, Child neglect, Emotional abuse, Web-based application, Parent education

## Abstract

**Background:**

Child abuse is one of the major health and social problems in the world and has severe short-term and long-term consequences on children’s psychological, social and physical functioning. One of the effective strategies to control and prevent child abuse is training parent through web-based applications. The aim of this study is to design and evaluation of child abuse web-based application for parent education and strengthen.

**Methods:**

This study is an applied-developmental study that performed in Razi Educational and Therapeutic Center in Tabriz. The study consisted of three main phases. The requirements assessment and design phases were completed between November 2022 and February 2023. The research community was parents referring to Razi Center and convenience sampling was used to select the samples. In firststage, a questionnaire was designed by searching in library sources and consulting with specialists for needs assessment and application design. The questionnaire was completed by psychiatric specialists, health information management and health information technology.Finally, the usability of designed application was evaluated with the participation of 30 parents and specialists.

**Results:**

Based on the identified information elements and capabilities, a child abuse web-based application was designed. Application capabilities were such as concepts of child abuse, prevention and treatment strategies, parenting skills, childrens behavioral disorders, child abuse laws and interaction with clinical specialists. Finally, the result of the web-based application usability evaluation was evaluated at a good level equal to an average of 7.6 out of a total of 9 points.

**Conclusions:**

The possibility of expressing experiences, exchanging message, attractiveness, ease of use, and accessibility of parents, they were designed as application features. The usability of the web-based application was satisfactory to users in various of overall functionality, display, terminology, learning ability and overall application capability.

## Background

Children are one of the most vulnerable groups in society and are constantly exposed to all kinds of dangers [1]. One of these threatening dangers is child abuse and despite the efforts of organizations supporting child right, this phenomenon is still increasing in the world [2]. According to World Health Organization(WHO), child abuse is harm and threat to the physical, mental and well-being of a child under the age of eighteen by parents, people close to the child, family or strangers [3].

Although there are similarities and differences in the definitions of child abuse, according to various sources, there are generally four types of child abuse: sexual abuse, physical abuse, emotional abuse and neglect [4]. Sexual abuse is considered as sexual violence against a child that forces the child to participate in sexual activities and pornography [5]. Physical abuse refers to non-accidental behaviors resulting in physical harm to the child (such as hitting and pulling hair) [6]. Emotional abuse includes behaviors that make the child feel worthless and unloved (such as verbal threats, teasing, and yelling) [7]. Neglect means insufficient care of the child’s physical and emotional needs (such as not providing proper clothes, food, and hygiene) [8].

The WHO has defined the main causes of child abuse in three groups: personality factors of the abuser, the characteristics of the abused person, and social, economic and cultural reasons [3]. There are many personal and social reasons that increase the risk of child abuse: child age, gender, physical or mental disability of the child, young age of parent, drug addiction of parent, low level of education of parents, lack of familiarity with parenting skills by parent, poverty, orphaned children and lack of social support [9].

Child abuse has many short and long-term consequences on the child and may continue throughout the child’s life in the form of various complications [10]. In addition, child abuse may lead to depression, mental disorders, physical aggression, antisocial behaviors, post-traumatic stress disorder (PTSD), low self-esteem, and suicide in adulthood. This phenomenon can impose irreparable economic and social costs on society [11].

The WHO has estimated that every year approximately 50 million children between the ages of 4 and 10 worldwide suffer from child abuse [12]. According to the United States Department of Health and Human Service (USDHHS), the number of children who experienced child abuse fluctuated from 656,000 to 674,000 between 2013 and 2017, an increase of 2.7%. The data of this department in 2018 shows that 74.9% of these children were exposed to neglect, 18.3% to physical abuse and 6.8% to sexual abuse [13].

In Iran, due to cultural and social issues, there is no accurate statistics on the prevalence of child abuse [14]. Unofficial statistics indicate the prevalence of this phenomenon in Iran and these statistics are reported through social emergency. In this way, in 2018, almost 14,000 calls related to child abuse were reported to the social emergency system and after verification, the occurrence of child abuse was confirmed in about half of them [15]. Child abuse in Iran ranks first among domestic violence and daily 20 to 25 cases of child abuse are registered by social emergency [16]. While it seems that the real statistics are more than the reported statistics because most families refuse to report child abuse and women are afraid that after reporting the abuse, they will suffer more violence from their husbands [17].

The family is one of the main factors in child care, and many specialists consider parent training to be the most important measure to control and prevent child abuse [18]. Parent training aimed at reducing abnormalities and abusing behaviors can have positive consequences because there is a multidimensional relationship between parents and the child’s health status [19]. Also, child development is one of the most important responsibilities for parents and the lack of awareness and knowledge to control and prevent this phenomenon may lead to many harms such as all kinds of abuse [20]. Parent training can lead to reforming educational patterns, strengthening parenting skills, and as a result, controlling and preventing all kinds of abuse and its consequences in childhood and adulthood [21].

One of the main interventions in child abuse is parent education. Parent education can be very useful in reducing behavioral abnormalities and child abuse behaviors because there is a strong relationship between parents and their children’s health. Many child and adolescent psychiatry experts consider parent training to be the most important measure to reduce child abuse behaviors. Parent education can lead to the correction of educational patterns, control and prevention of various injuries to children, and finally control and prevention of severe consequences of child abuse in childhood and adulthood. And consequently prevent the transmission of harm to the family and society [22].

Today, information technology is an important factor that provides countless opportunities and benefits such as saving money, time and the possibility of accessing information [23]. The use of information technology in the field of disease control and prevention has been able to help people to overcome many challenges and limitations such as lack of access to information, dispersion of information, face-to-face visits, distance from medical centers and cost of care services [24]. In recent years, the use of these technologies has increased dramatically especially web-based applications [25]. According to the report published by the Institute of Health and Medical Informatics (IHMI), until 2018, approximately 231,000 applications have been made available to consumers, and the amount of use of such applications is increasing significantly [26].

An application is a tool that supports processes to help solve semi-structured or unstructured problems by providing different options [27]. Web-based applications with features such as user-friendly interface, innovation in education, flexibility and easy access have caused many health specialists to have a great desire to use these applications to provide care services [28].

The main focus of care models in the field of child abuse is based on the role of parent and moves towards activities that are managed by parent [29]. The combination of these models and information technology can play an important role in controlling and preventing the phenomenon of child abuse [30]. Considering the role of web-based applications in facilitating the education of parents and increasing their awareness of various aspects of child abuse, the purpose of this study was to. design and evaluation of a child abuse web-based application for parent education & strengthen.

## Methods

. This project was an applied-developmental study that development phase performed between November and December 2022 and evaluation phase performed between January and February 2023 as a part of the CHATR (Child Abuse and Trauma Research) project. The CHATR project is a program developed at the Tabriz University of Medical Sciences in the field of comprehensive management of child abuse at individual, family and social levels. This study was approved by the ethics committee of Tabriz University of Medical Sciences ( IR.TBZMED.REC.1400.344).

**Fig. 1:**
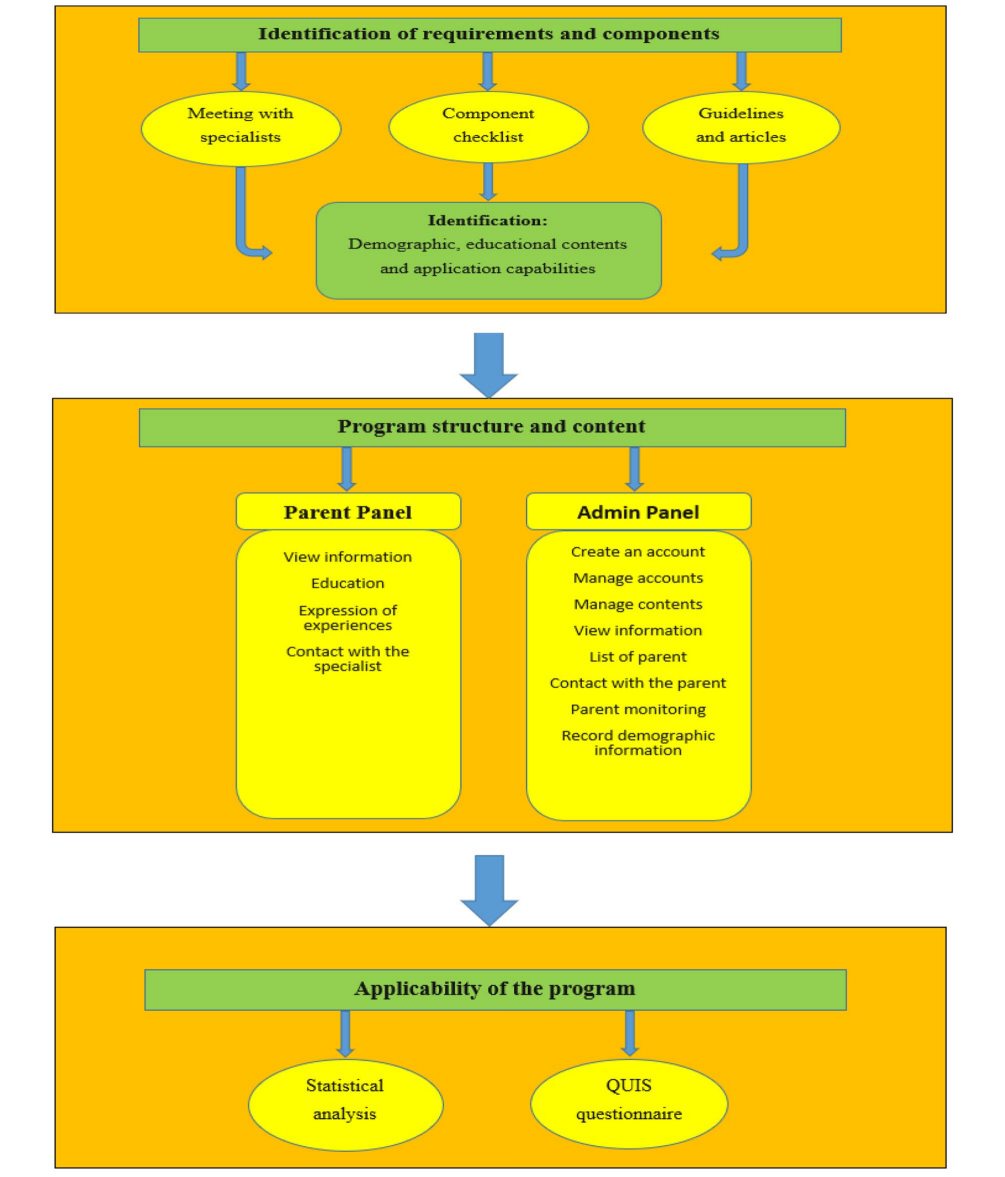
The schema of methodology

The study community included specialists in child and adolescent psychiatry, health information management, health information technology and parent referring to the Razi Educational and Therapeutic Center in Tabriz. This center is the largest hospital in the field of psychiatry in East Azerbaijan province. The research community was parents referring to Razi Center and convenience sampling was used to select the samples. The 5 specialist included experts in psychiatric specialists, health information management and health information technology. The specialist and parents coordinatd in design and evaluation phase of system… The 30 parents who had sufficient ability to use the web-based application and had sufficient e-health literacy and also had the desire to participate in the study were selected as a sample. Parents who lacked the ability to read, write and use the web-based application were excluded from the study. Also, parent provided informed consent before inclusion in our study. This study has been performed in three steps including formulation of the required components, application design and evaluation. The flowchart of the proposed application is shown in Fig. 1.

### Step 1: develop effective requirements and capabilities in the child abuse web-based application

The first step of needs assessment for designing educational software and applications is to identify information needs, effective components and expectations from the application.

The capabilities, content, user and features of the application, as well as the needs it must meet, should be reviewed.

This web-based application required data elements and capabilities were identified and extracted with a comprehensive review of the articles, guidelines and valid databases. Then to better identify the needs of system users and experts, a questionnaire was prepared according to result of studies. Database such as PubMed, Scopus, Web of Science, Science Direct and Embase were searched to identify and select required data elements and capabilities. The search strategy utilized keywords such as “child abuse,” “child maltreatment,” “child neglect,” “emotional abuse,” “physical abuse,” “sexual abuse,” “telemedicine,” “telehealth,” “telecare,” “skills for parents,” “parenting skills,” “prevention of child abuse,” child support organizations,” “disorders related to children,” “therapeutic interventions,” and “application”. The keywords were combined using Boolean operators of “AND” and “OR”.

A variety of studies were considered to find web-based application educational content and extract all existing efforts to prevent and managing child abuse including randomized controlled trial (RCT), case control, experimental, pre and posttest, cross-sectional and mixed methods studies. finally, we reviewed the reference lists of studies identified for inclusion. The search strategy was designed with the assistance of two information management and content experts. One author (S.P) performed the literature search and two information management specialists (Z.M and E.M) confirmed the correctness of the search strategy. Two independent authors screened titles and abstracts resulting from all searches to identify potentially eligible studies. A full-text review of each study was then performed by two independent authors; A third independent author settled any discrepancies. Finally, studies were selected that were in the field of parent education, prevention and treatment of child abuse. Selected studies were in English language and studies in other languages were excluded. Selected studies included two categories. The first category of studies were related to the extraction of educational content related to child abuse (which includes: familiarity with types of child abuse *n* = 7, parenting skills *n* = 5, behavioral skills for parents *n* = 8, organizations supporting children’s rights *n* = 3, prevention and treatment of child abuse *n* = 11, Mental and behavioral disorders of children *n* = 6). Also, the second category of studies were related to determining the functional capabilities of the web-based application *n* = 17. Selected studies were in English language and studies in other languages were excluded. By studying the selected studies to extract educational needs, 84 titles were extracted and after review by child psychiatry specialists, they were summarized in the form of 56 questions to be included in the need assessment questionnaire.

By summarizing, integrating and prioritizing the extracted requirements, as well as consulting with specialists in child and adolescent psychiatry, a questionnaire was designed with 56 questions on a 5-point Likert scale (agree strongly to disagree strongly). The questionnaire consisted of three main sections: demographic information (9 questions), educational content information (39 questions) and web-based application capabilities (8 questions). Also, at the end of each section of questions, an open-ended question was considered to add the items by specialists. The Face validity of the questionnaire was confirmed by three child psychiatrists and two health information management specialist. At these stage experts scored questions on simplicity, clarity and relevance to the objectives. Items were selected whose impact factor was above 1.5. The reliability of the questionnaire was calculated by Cronbach’s alpha 0.93..

Then specialists in child and adolescent psychiatry, health information management and information technology specialists commented on the requirements of the web-based application in the form of a researcher-made questionnaire. The questionnaire was given to specialists in person and in some cases, through social networks and collected by them after completion. The results of the survey were analyzed using descriptive statistics and frequency distribution report in Spss software. Based on the scores given by participants in the study, the frequency and average scores for each data element were calculated. Then, considering that the highest possible score for each data element was 5, half of this was considered an average score for each data element. As a result, each of the data elements was considered essential if it obtained at least an average of 2.5 according to the specialists opinion. Also, if a new data element was suggested by at least 50% of the participants in the open-question section of the questionnaire, the data element would be used in the design of the web-based application.

### Step 2: design the content and structure of the child abuse web-based application

Based on the identified capabilities in the first step and meetings held with information technology specialists, the ASP.NET Core programming method was selected in the Microsoft Visual Studio 2019 software and SQL server 2016 database to develop the web-based application. ASP.NET Core is a framework based on C# programming language, which was produced by Microsoft to create web services and web-based applications, and it works much better than other frameworks in terms of speed, security and application execution.

The basic structure of the web-based application was designed in the form of two separate panels for admin and parent. In user panel, parents had access to capabilities such as viewing educational content, exchanging message with child psychiatrists, and editing demographic information. In addition to these, in the admin panel, capabilities such as managing educational content(such as add new content, edit and delete)g, new user registration and application dashboard management were available. Considering cultural issues in Iran and limitations in expressing some topics related to sexual abuse, we tried to localize the design of web-based application on the cultural characteristics of the participants. For example, during the production of educational content, some educational posts extracted from external sources in the field of sexual abuse which were contrary to cultural characteristics in Iran were removed. The application was used and its functional capabilities were evaluated. According to evaluation result, the most modifications were related to the use of attractive user interface and add search ability to application,.

In designing of web-based application, criteria such assimplicity, accessibility and interaction between the parent and the psychiatry specialist were considered. One of the advantages of the designed application is its responsiveness to different hardware. Responsiveness means that if the user enters the panal page of the application using smartphones or computers, the desired page will take a special shape for each of these devices in relation to its size.

### Step 3: evaluate the usability of the child abuse web-based application

After designing a web-based application, with the provision of initial training and sufficient explanations by the researcher during four training sessions, the application was made available to parent referring to Razi educational and therapeutic center. At the same time, the application were available to specialists. The selection of patients was done by convenience samplingso that users should have a laptop, computer or smartphone and have sufficient ehealth literacy. The child abuse web-based application was used by 30 parent for at least 30 days. Then, participants opinions about the usability of the application were gathered by using the standard usability and user satisfaction assessment questionnaire (QUIS) [31]. The questionnaire was given to parent and specialists in person and in some cases through social networks and collected by them after completion. This questionnaire for user interface satisfaction elicits user opinions and evaluates user acceptance for computer interface. As a standard questionnaire, QUIS includes 27 questions related to the evaluation of usability and user satisfaction. This questionnaire consists of five sections: overall software performance, terminology and information, learning and (6 questions), screen (4 questions), software terminology and information (6 questions), learning (6 questions) and general impressions (5 questions). Each question has an answer with a score of zero(minimum satisfaction) to nine (maximum satisfaction). A score of 0 to 3 was classified as a poor level, 3 to 6 as a moderate level, and 6 to 9 as a good level. Data were analyzed using descriptive statistics (mean, variance and standard deviation) in spss software.

## Results

Information elements, educational contents and capabilities of the web-based application were extracted from a review of sevaral articles related to the research. The demographic characteristics of the specialists are described in Table 1. The results of the survey showed that all the elements identified valuable from the specialist’s point of view and were recognized as essential. Among the demographic information of parent, age, gender, marital status and occupation scored the most points in the survey. In the educational content section, knowledge of the types of child abuse, consequences of abuse, prevention methods and parenting skills were recognized as absolutely necessary by all participants (score 5). In the web-based application capabilities section, by surveying health information technology and health information management specialists, all the identified elements had a higher than average value. In this section, elements related to capabilities such as application web-based, information exchange and separate panels for parent and admin received the most points (score 5).

**Table 1:** Demographic information of specialist in the needs assessment stage

Gender		Age (Year)		Education		Experiences	
Man	60%	20–30	20%	Child and adolescent psychiatrist	30%	0–5	10%
Woman	40%	30–40	35%	Social doctor	30%	5–10	20%
		40–50	35%	Health information technology	20%	10–15	40%
		50–60	10%	Health Information Management	20%	15–20	20%
						20–25	10%

**Fig. 2:**
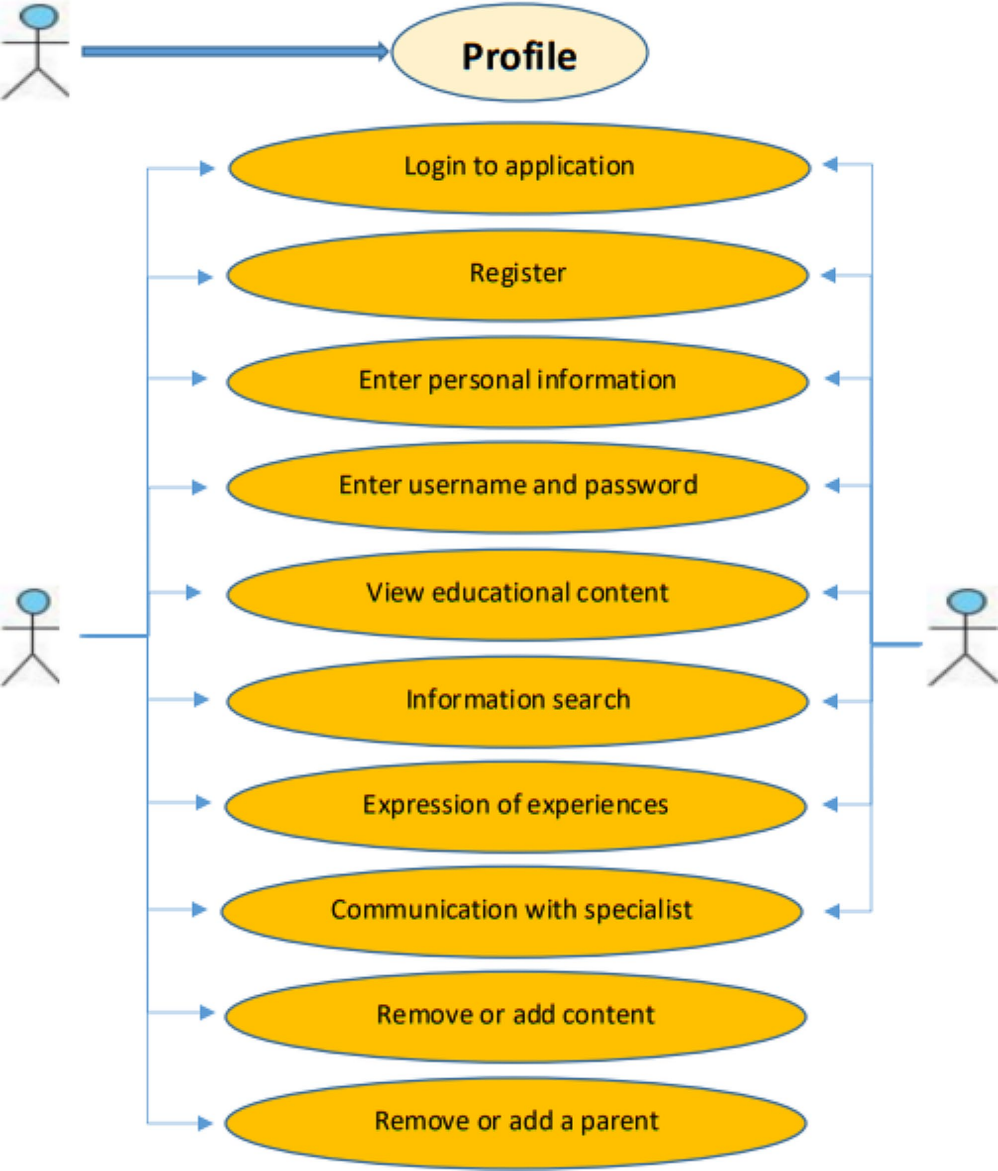
User case diagram of proposed child abuse web-based application

According to the identified requirements and capabilities in the first step, a use case diagram to show capabilities and web-based application model was drawnby IBM Rational Software Architect software and UML language (Fig. 2). The child abuse web-based application was designed using Microsoft Visual Studio 2019 software and SQL server 2016 database in two separate panels for admin and parent.

**Fig. 3:**
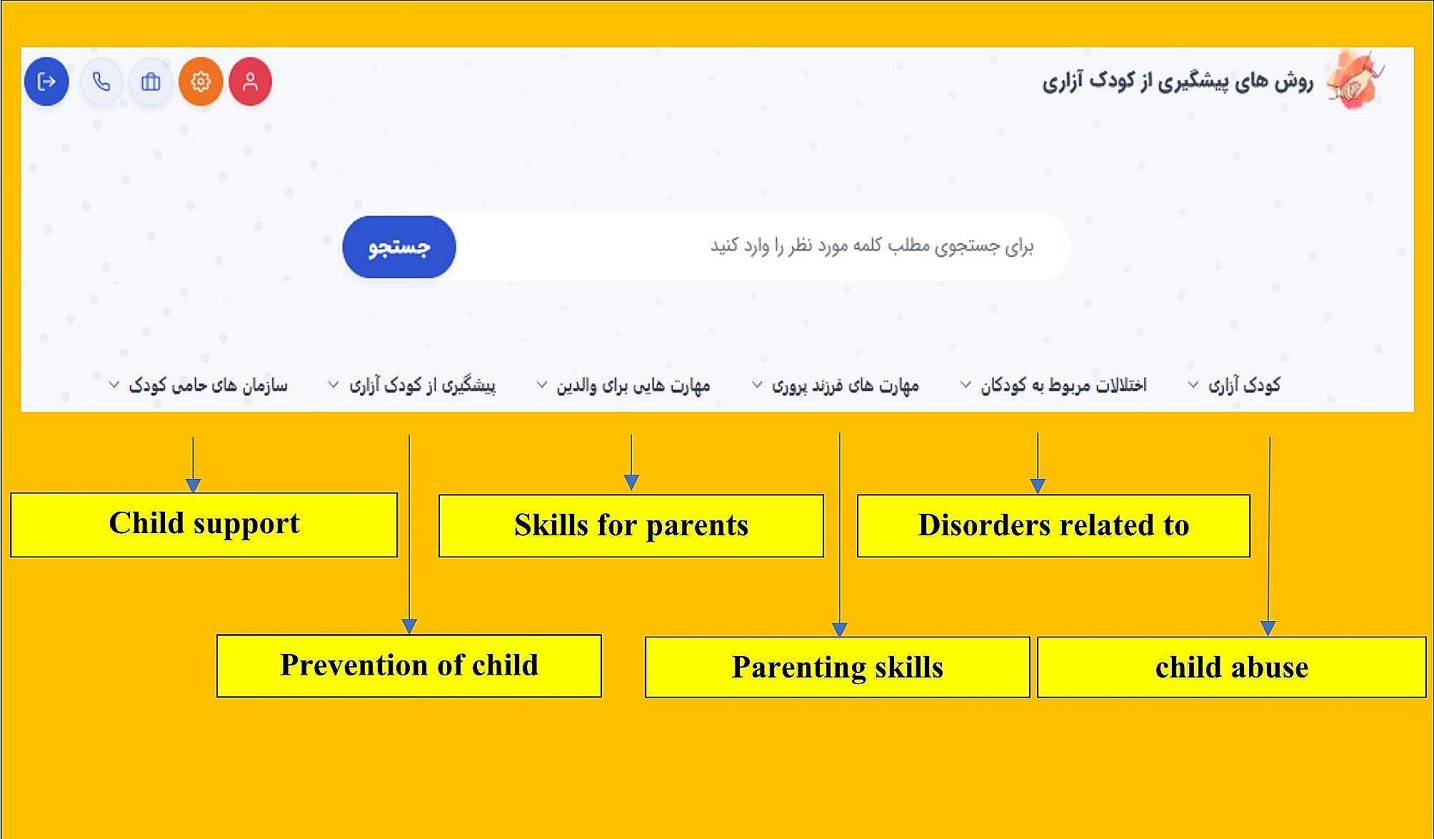
The schema of parent panel

**Fig. 4:**
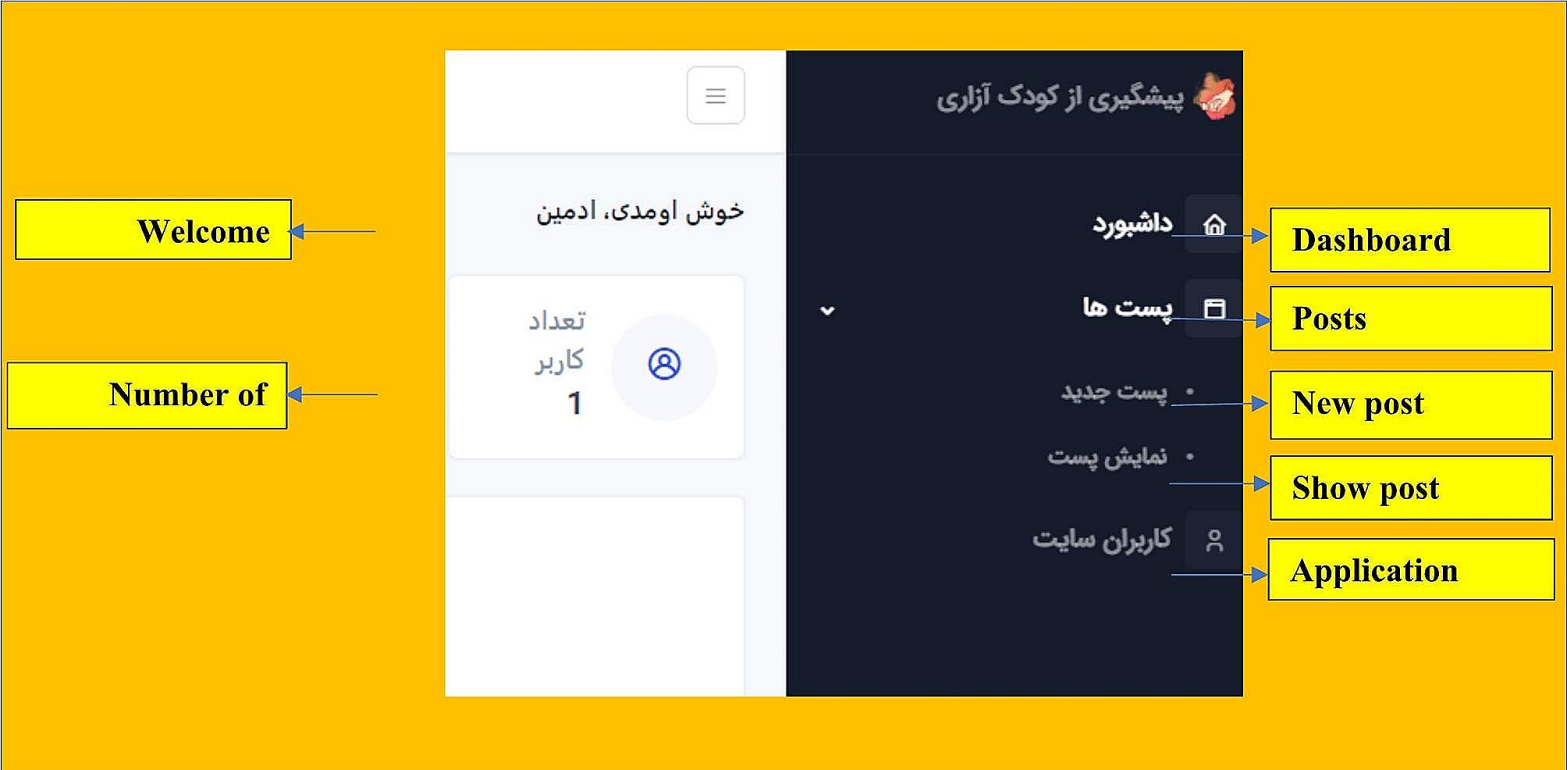
The schema of specialist panel

After sampling and receiving informed consent from participants, registration of specialists and parents was done by the researcher. This web-based application has two separate panels for parent and a child psychiatrist. The parent panel is the main part of this application, and parent who wanted to participate in the study could access this panel and its educational contents after registering and defining a username. After entering the user name by the parent, they will be directed to the main page of the web-based application and gain access to educational content such as familiarity with child abuse, children behavioral-psychological disorders, parenting skills, behavioral skills for parent, prevention of child abuse, organizations supporting child rights and child abuse laws in Iran (Fig. 3). The web-based application in the parent panel provides capabilities such as viewing educational content, editing personal information, expressing experiences of child abuse and communicating with a child psychiatrist. In addition, after being registered by the researcher, the child psychiatrist had access to capabilities such as viewing the list of registered parent, adding new parent, viewing educational content, exchanging opinions through the chat room designed in the application and determining time to visit in person if requested by parent (Fig. 4).

**Fig. 5:**
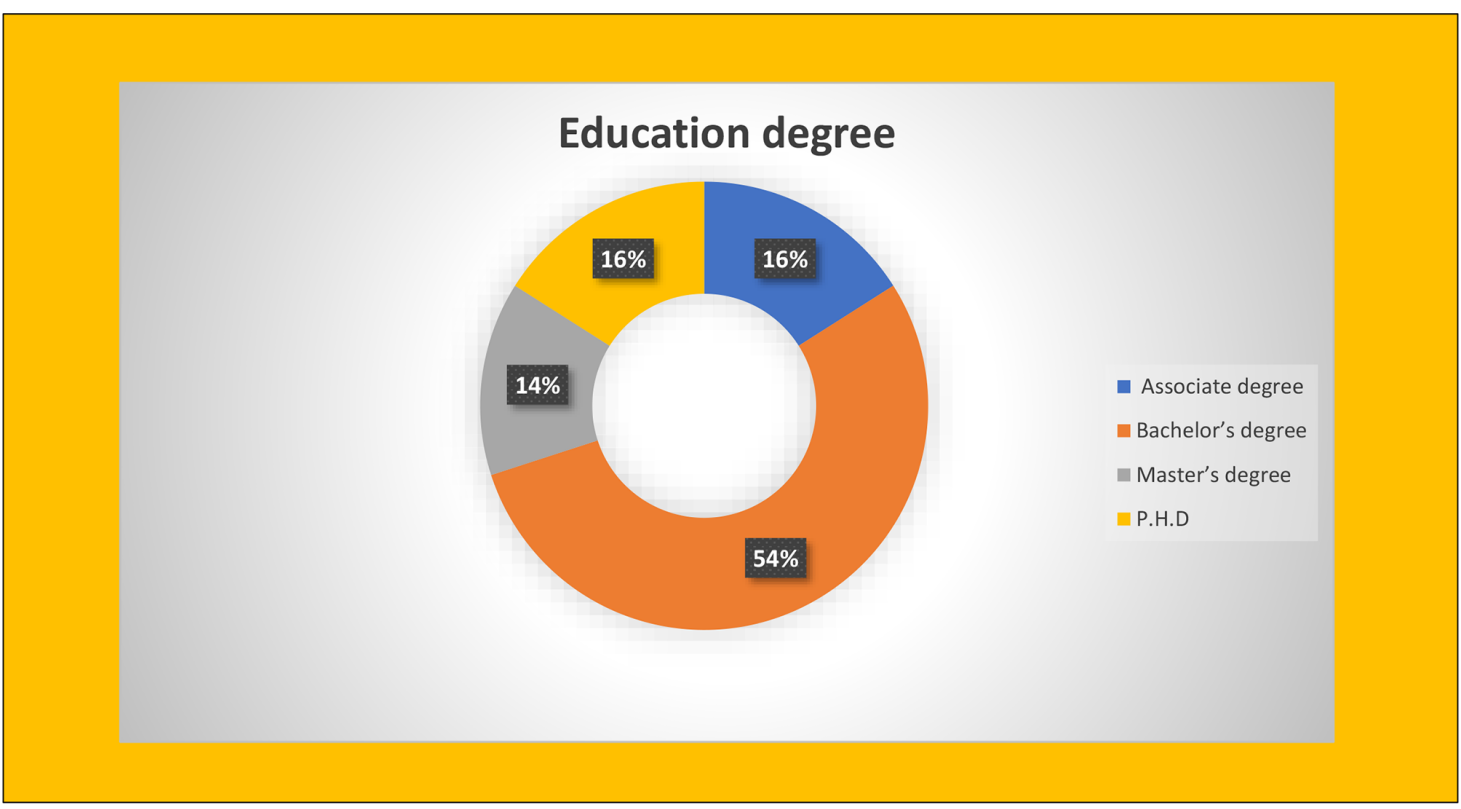
Frequency distribution of all participants’ educational level (

**Fig. 6:**
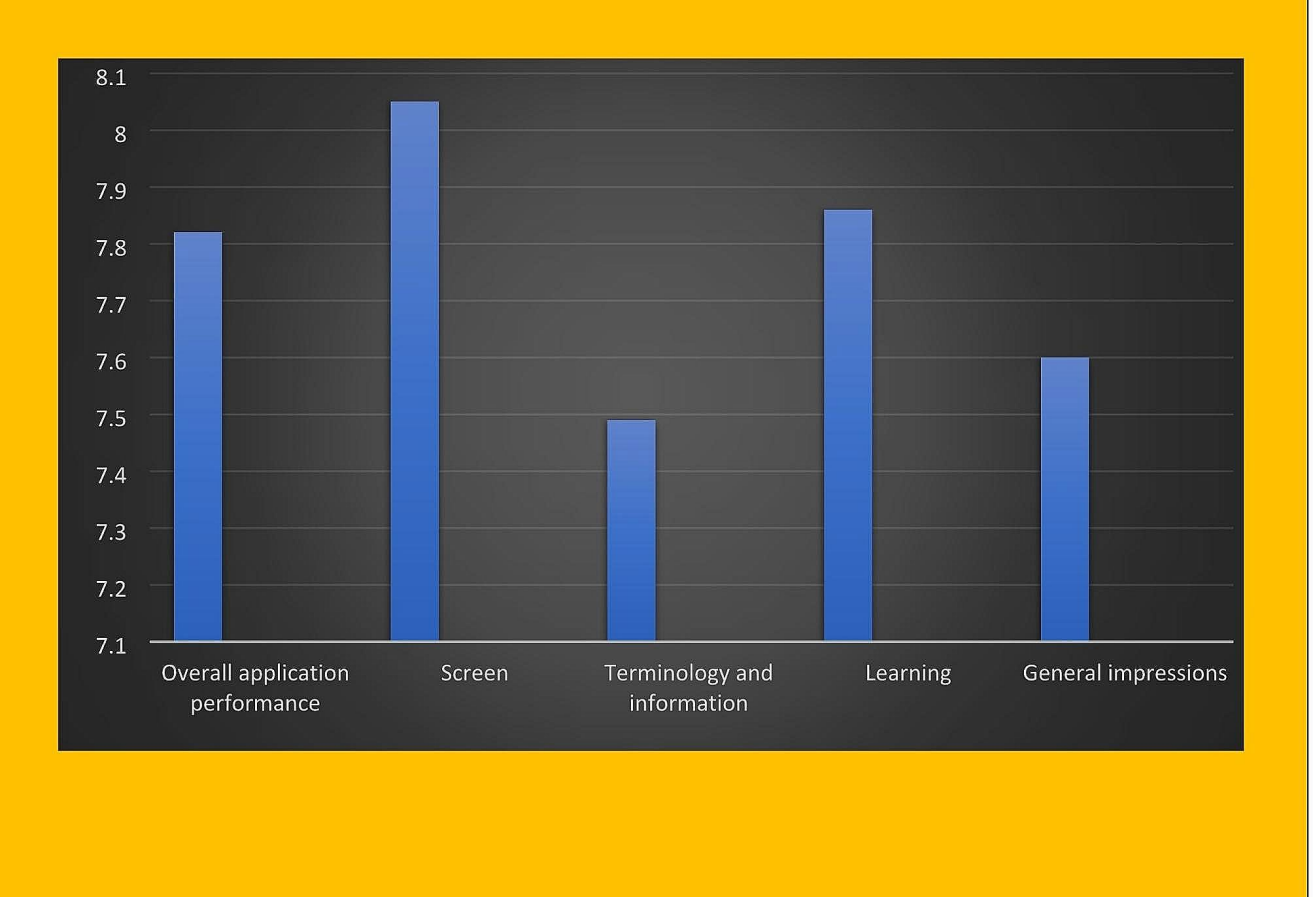
Average system usability scale scores

With the participation of 30 parents and the cooperation of the Razi Educational and Therapeutic Center in Tabriz, the web-based application was evaluated using a standard questionnaire to assess the usabilityand satisfaction of QUIS participation. Among the 30 participants in the present study, men (60%) were morelikely than women (40%) in terms of gender. The majority of participants were in the age range of 30–40 years (40%) and 40–50 years(30%). 55% of the participants had a bachelor’s degree. The results of the study showed that the education level of the participants was directly related to their willingness to participate in the study. Because the participants who had a higher education degree were more willing to participate in the study, and unlike them, the participants who lacked e-health literacy refused to participate in the study due to their inability to use the web-based application and read the educational content.The frequency distribution of the education level of participants is shown in Fig. 5. Questionnaire QUIS collected the participants’ views about the usability of the child abuse web-based application in five areas: overall application performance, screen, terminology and information, learning capability and overall application capabilities. The QUIS questionnaire was designed based on the nine-point Likert scale. Each question had an answer with a score of zero to nine. Scores were classified as follows: 1 to 3 as poor, 4 to 6 as intermediate, and 7 to 9 as good. The results of participants’ surveys were analyzed and evaluated using descriptive statistics such as mean, variance and standard deviation in SPSS software. According to the results obtained in Tables 2 and 3 as well as Fig. 6, the summary and general average of participants views on the applicability of the web-based application was 7.6 and evaluated at a good level.

**Table 2:** The result of participants’ viewpoint about usability and satisfaction of application

Features		Indicator		
		Average	Standard deviation	Variance
Overall application performance	General function of the application	8	0.44	0.64
	The difficulty of working with the application	7.96	0.48	0.9
	How do you feel about using the application?	8.26	0.57	0.69
	Overall application design	7.9	0.54	0.75
	Continuous work with application	7.73	0.84	0.91
	Application configuration capabilities	7.13	1.15	1.08
Screen	Readability of the letters on the screen	8.46	0.92	0.94
	Easily perform tasks with specific phrases	7.66	0.52	0.92
	Organizing information	8.16	0.86	0.84
	Screen sequences	7.93	0.95	0.92
Terminology and Information	Use terms in the application	7.9	0.92	0.94
	A set of terms related to working with the system	7.36	0.85	0.86
	Location of messages on the screen	8.3	0.35	0.5
	Message to record essential data	7.56	1.04	1.06
	System messages regarding the completion of tasks	6.86	0.78	0.84
	Application error messages	7	1.16	1.08
Learning	Learn to work with application	8.2	0.41	0.58
	Discover application features with trial and error	8	0.81	0.89
	Save names and use features	8.13	0.95	0.92
	Perform tasks quickly and easily	7.13	0.8	0.83
	On-screen help messages	7.63	0.75	0.82
	Application usage guide	8.1	1.25	1.35
General impressions	Application speed	8.1	0.48	0.68
	Application availability	7.7	1.25	1.43
	Multiplicity of application capabilities	7.3	0.47	0.64
	Correction of user errors	7.3	0.79	0.82
	Design to suit different users	7.63	0.72	0.81

**Table 3:** The overall evaluation of the application usability and satisfaction

Phrase	Average	Standard deviation	Variance
Overall application performance	7.82	0.67	0.82
Screen	8.05	0.82	0.91
Terminology and information	7.49	0.85	0.88
Learning	7.86	0.82	0.90
General impressions	7.60	0.75	0.88
Average	7.76	0.79	0.88

## Discussions

In the present study, the requirements of web-based child abuse application for parent education were identified in three main areas: demographic information, educational content, and application capabilities. The present study is similar to the studies performed in the field of designing educational applications [32]. In similar studies with the goals of designing applications for AIDS and heart failure, in the first step, demographic information, educational content and application capabilities were identified [33, 34].

Gulirmack et al., a study aimed at effectiveness of web-based distance education for parent in the prevention of emotional abuse and neglect, categorized the information needed to design web-based application into seven sections: familiarity with child abuse, mental health in children, parental behavioral characteristics, reward and punishment for children, parenting skills, and parent demographic characteristics [35]. According to the approach they pursued to identify educational content, the present study collected educational content in seven sections: familiarity with child abuse, child behavioral disorders, parental behavioral skills, parenting skills, child abuse prevention, child rights protection organizations and child abuse laws in Iran. In addition, the present study enabled registration to enter new parent and use educational content.

Caroline et al., a study aimed at identifying the standard components of parenting programs to prevent child abuse, identified fourteen evidence-based parenting programs using online database reviews related to evidence-based programs identified [36]. According to the Caroline study strategy, in the present study, parenting programs were also extracted from valid databases and were placed as educational posts in the child abuse web-based application for parental education.

In the application capabilities section, reviewed studies had similar approaches to selecting the application capabilities. In the study of Baggett et al., the web-based application designed for child abuse prevention had capabilities such as defining a username and password, consulting and exchanging messages, searching for information, and adding new participants [37]. In this current study, using the approach of Baggett et al., similar functional capabilities were considered for the child abuse web-based application, which includes: consultation and message exchange between specialist and parent, defining username and password, possibility to change password, being web-based, adding new users, application guide for parent, separate panels for specialist and parent, information search.

Various studies have been performed to evaluate the usability of mhealth applications. Because the issue of usability and its factors is a well-known factor in determining the success and acceptance of software and IT technologies, which has received much attention.

Zapata et al., in a systematic review study, divided usability into attractiveness, learning, performance,and comprehensibility according to the ISO 9126-1 quality model [38]. In the present study, participants rated attractiveness 8.5, learning ability 7.86, efficiency 7.82, comprehensibility 7.49 and the application was evaluated at good level.

In the study of Yasini et al., “usability” included ease of use, performance, readability, information needs, completeness, flexibility, user satisfaction, and good feeling of participants after using the application [39]. In the present study, the criteria were evaluated as follows: ease of use 7.96, readability 8.46, information needs 7.49, flexibility 8.05, user satisfaction 7.82, completeness 7.6, and the user good feeling after use 8.26.

In another study, Brown et al. described the health information technology evaluation model (Health-ITUEM) to answer the gaps in the application models that were previously developed. Health information technology evaluation model was developed as a comprehensive usability evaluation framework. The criteria of this evaluation model include: error prevention, completeness, recall, information needs, adjustability, learning ability, performance speed, competency and other results [40]. The average participants comments of the child abuse web-based application designed during this research were for error prevention 7, completeness 7.6, recall 7.56, information needs 7.49, adjustability 7.13, learning 7.86, performance speed 8.1, competency 7.6 and were evaluated at a good level.

One of the educational items that was highly regarded by specialists in the needs assessment phase was how to report child abuse cass observed by parent. The issue of what actions parent should take when they observe cases of child abuse by neighbors or other people outside the family environment was one of the educational items that was included in the child abuse application based on the advice of specialists. A number of identified studies and software application were designed to teach the principles of reporting child abuse cases observed by parent or child care providers (CCPs) to child rights organizations [41, 42]. The effectiveness of educational interventions using an online software application to improve the knowledge and attitudes of parent and CCPs about reporting cases of child abuse was investigated in a study by Yang et al. Educational content was provided to parents and CCPs using an online and interactive learning module about reporting child abuse. The results of the study showed that in a virtual environment, improved knowledge and attitudes of parent and CCPs about reporting cases of child abuse [43].

In this study, many parent stated that they do not have enough knowledge and information in the field of reporting cases of observed child abuse and organizations that support child rights. In the web-based application designed in this study, the principles of reporting child abuse and familiarization with organizations and institutions supporting child rights in Iran, such as social emergency, were placed in the form of educational posts for parent. One of the parents said, “getting to know these organizations and reporting cases of child abuse can play a special role in reducing and preventing all types of child abuse”. The web-based application designed in this study, like the studies performed, by providing the necessary training, led to the satisfaction and improvement of parent knowledge in the field of reporting child abuse and familiarity with organizations and institutions that support child rights.

The web-based application (iBA) was designed by obikane to improve depression symptoms and prevent neglect in mothers after postpartum. Although this web-based application led to improvement in postpartum depression symptoms for mothers, only depressive disorder was considered in this study. This is despite the fact that there are many mental and behavioral disorders that significantly increase the risk of child abuse, neglect and emotional abuse by affecting the behavioral performance of parent [44].

The application designed in this study, compared to the obikane application, in addition to covering depression disorder, includes educational posts about various disorders such as hyperactivity, oppositional defiant disorder, autism, stress, anxiety, personality disorders, mental retardation, abnormal fears and many other disorders. The possibility of communicating with specialist and expressing experiences is one of the other advantages of the application designed in this study.

Another important issue in the identified studies was the strengthening of parenting skills for parent [45, 46]. Training and strengthening parenting skills is one of the most basic items of applications designed in the field of child abuse [47]. Parent who have good parenting skills can use these skills to create a healthy and safe educational environment for the child and reduce the potential of committing child abuse behaviors to a significant extent. Some of these skills include: strengthening childr positive behaviors, how to solve children incompatibility with each other, how to raise a child healthy, how to strengthen a child self-confidence, how to managing behavior with an angry child, managing a child who makes excuses, managing children aggression, how to interact with a child informant, story therapy, etc. [48, 49].

In a study, Jennifer et al. examined seven families to strengthen parenting skills to reduce child behavioral disorders. Parent who received parenting skills interventions showed more positive parenting behaviors during play with the child than parent who did not receive the intervention [50]. In the present study, parenting skills were extracted from reliable scientific sources and provided to parent in the form of educational posts. Parent said that by learning these skills, they could significantly overcome many problems and incompatibilities created between themselves and their children with each other. In this study, parent were willing to learn more about child abuse prevention interventions and parenting skills to prevent child abuse. Also, most of the messages exchanged in the chat box space of the application emphasized this issue. Mhealth technologies could provide a great opportunity to educate parents about child abuse. Most parent believed that the use of these technologies could significantly improve their awareness and knowledge in the field of child abuse without the need for in-person referrals to psychologists and counselors.

## Conclusions

The use of mhealth technologies, especially web-based applications, are recommended as a tool for training parent, strengthening parenting skills, access to information, control and prevention of child abuse. In recent years, educational applications have been developed for the management of various diseases. The special nature of the phenomenon of child abuse and the possibility of committing all kinds of abuse by parent, clarifies the importance of paying special attention to design and develop appropriate solutions in order to provide proper education, modify behavioral styles, counseling, improving knowledge and awareness.

In the designed web-based application, it was tried to pay attention to all kinds abuse: sexual, physical, emotional and neglect. The possibility of expressing experiences and communication between parents and psychiatric specialist in the form of chat was one of the unique features of the web-based application. Also, one of the other benefits of this web-based application was familiarization with types of child abuse, prevention and treatment methods, parenting skills, child behavioral disorders, organizations supporting child rights, and child abuse laws in Iran.

The results of this study showed that the designed child abuse web-based application has been able to satisfy users. From the participants’ viewpoint, the usability of the child abuse web-based application was evaluatedat a good level with an average score of 7.6 out of a total nine points. The usability of the child abuse web-based application in various areas of overall functionality, display, terminology, learning capability and overall application capability was satisfactory to participants.

## Data Availability

The data that supported the findings of this study are available from the corresponding author on request.

## Abbreviations

AIDS: Acquired immunodeficiency syndrome
CCP: Child Care Provider
CHATR: Child Abuse and Trauma Research
IHMI: Institute of Health and Medical Informatics
ISO: International Organization for Standardization
ITUEM: Information Technology Usability Evaluation Model
mHealth: Mobile Health
PTSD: Post-Traumatic Stress Disorder
QUIS: Questionnaire for User Interaction Satisfaction
SQL: Structured Query Language
UML: Unified Modeling Language
USDHHS: United States Department of Health and Human Services
WHO: World Health Organization
